# Foreign bodies in the rectum: Three case reports of sexual violence

**DOI:** 10.1016/j.amsu.2022.103695

**Published:** 2022-04-29

**Authors:** Arash Mohammadi Tofigh, Sohrab Salimi, Behzad Nematihonar, Javad zebarjadi Bagherpour, Fawzia Negin, Pashton Qaderi

**Affiliations:** a Department of Surgery, Imam Hossein Hospital, Shahid Beheshti University of Medical Sciences, Tehran, Iran; b Department of Anesthesiology, Imam Hossein Hospital, Shahid Beheshti University of Medical Sciences, Tehran, Iran; c Faculty of Medicine, Balkh University, Balkh, Afghanistan; d Department of Psychology and Educational Sciences, Balkh University, Balkh, Afghanistan

**Keywords:** Sexual violence, Foreign body, Rectum

## Abstract

**Background:**

Sexual violence is one of the worst forms of violence with long-term physical and psychological effects on victims. It has been stated that sexual stimulation was responsible for 78% of clinically relevant foreign rectal bodies. About 10% of the cases were due to sexual assault. A problem commonly encountered in patients with RFB is the delay in presentation. While patients may be reluctant to disclose the cause of their presentation.

**Cases presentations:**

All the patients were males with a mean age of 41.1 years old. On average, they presented 2 days after the rape, Diagnosis was made in all 3 patients with a history and abdominal x-ray.The cause of the foreign body in each patient was violence and retaliatory behavior. Foreign objects included bottles, lamps, and water pipes. In 2 patients the foreign bodies were removed through *Trans*-anal procedure and in one patient laparotomy and colostomy need to be done for removing the Foreign object.

**Conclusion:**

Despite the urgency in the treatment of these patients, which involves the removal of a foreign body, special attention should be paid to psychological trauma and its long-term effects on patients' wellbeing. In stable, non-perforated patients, tans-anal approach under sedation is a good approach. If it fails, the patient needs to go to operating room for further anesthetic and surgical interventions.

## Highlights

• In stable, non-perforated patients, tans-anal approach under sedation is a good approach.
• Special attention to psychological trauma and its long-term effects on patients requires long-term follow-up.
• Rigid proctoscopy or flexible sigmoidoscopy is recommend after the rectal FB removal.

## 1 Introduction

Sexual violence is a major global health issue and it has been recognized as a public health concern by human rights entities as well as by international organizations, such as the world health organization (WHO). Rectal Foreign Bodies (RFBs) have always been one of the most sensitive and controversial topics in forensic medicine and its surgical management [1]. There is no reliable data about the incidence of clinically significant rectal foreign bodies, and the incident rate is significantly higher for men than women. In a systematic review study in 2010 a ratio of 37:1 was obtained between men and women, and the mean of ages was 44.1 years with a standard deviation of 16.6 years [2]. There are many reasons behind the insertion of a rectal foreign body including, criminal assault, self-treatment, sexual gratification, and even the occasional accidence [3,4]. But in most cases they are of sexual or criminal motivation. According to one study, sexual stimulation was responsible for 78% of clinically relevant foreign rectal bodies. About 10% of the cases were due to sexual assault [5,6]. The delay in seeking hospitalization is a major problem. While patients may be reluctant to disclose the cause of their presentation, diagnosis can be easily made with accurate history and confirmed with radiographs. It is vital to rule out signs and symptoms of peritonitis. It is always warranted to manipulated foreign body mannerly and if there is evidence of significant bowel injury or even perforation, surgical interventions will be considered [7]. Based on **S**urgical **Ca**se **Re**port, 2020 (SCARE) guidelines, here, we report the three cases of patients with RFBs who had been referred from forensic medicine with the purpose of surgical intervention [8].

## 2 Case presentations

### 2.1 Case.1

**Fig. 1:**
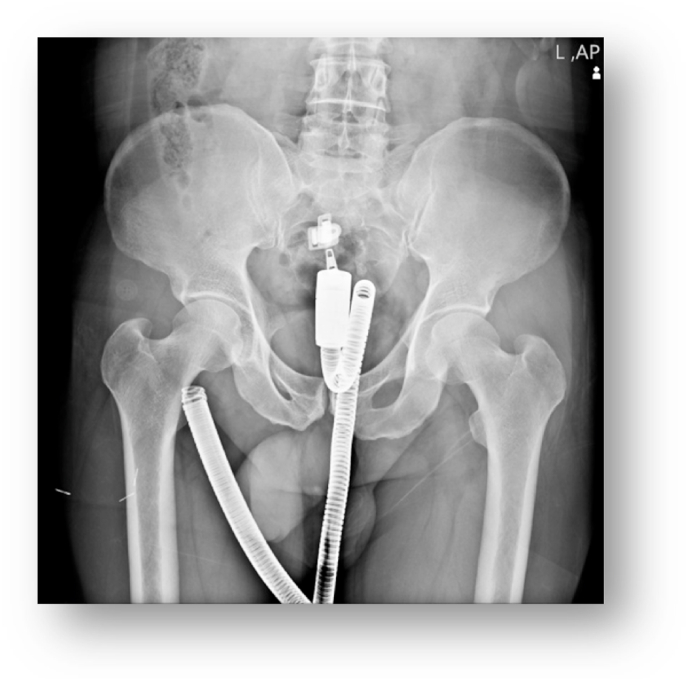
Intra-Rectal hose on abdominal plain film.

A 43-year-old male was referred to the surgical ward from the forensic medicine department for further surgical interventions. The patient was a case of sexual harassment after the conflict using the water hose two days before visiting the surgery ward. The patient complained of mild to moderate pain in the lower abdomen, and difficulty in defecation. On physical examination, there was a brief tenderness in the hypogastric region and superficial abrasions were seen around the anus. The history of sexual dysfunction, psychosocial, and drug history were unremarkable. With Plain radiographic plus strong forensic history (Fig. 1), the patient was taken to the operating room. Under sedation ((Midazolam, Fentanyl, Poopofol, and Lidocaine were administered for conventional induction. Meanwhile, Propofol was used for intraoperative maintenance as the dosage of 0.025–0.075 mg/Kg/Min), with the help of a speculum and pliers, the water hose had been removed successfully by the attending surgeon of the hospital.

### 2.2 Case.2

**Fig. 2:**
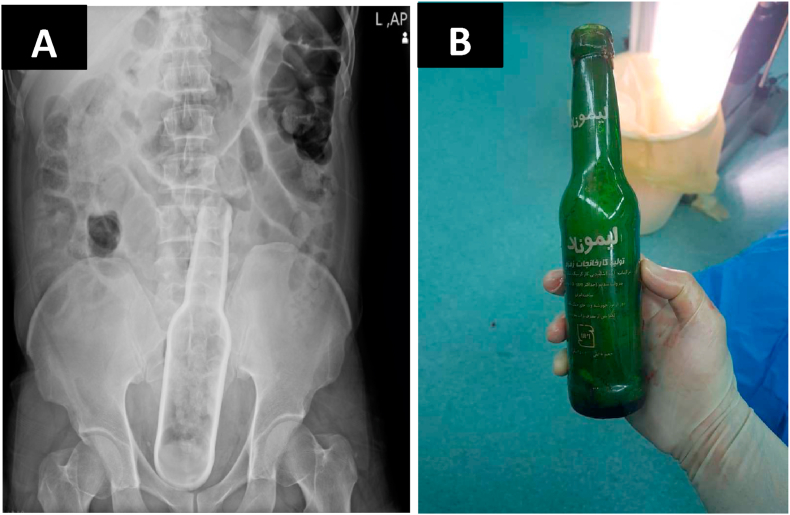
The images show (A), foreign body in rectum, and (B) the glass bottle after removal.

A 40-year-old man without a history of psychosocial was referred to the surgical ward after sexual violence that a glass bottle had been injected into the rectum. According to forensic history, the rectal foreign body has inserted following conflict and both sides of the conflict were brought to the police then forensic medicine. Upon arrival, the patient was complaining of lower abdomen pain and difficulty in defecation. On examination, there were abrasions around the anus and moderate tenderness in the hypogastric region. After Palin radiographic film the patient took to the operating room (Fig. 2). Under sedation (Midazolam, Fentanyl, Poopofol, and Lidocaine were administered for conventional induction. Meanwhile, Propofol was used for intraoperative maintenance as the dosage of 0.025–0.075 mg/Kg/Min) the foreign body had been removed by an attending general surgeon.

### 2.3 Case.3

**Fig. 3:**
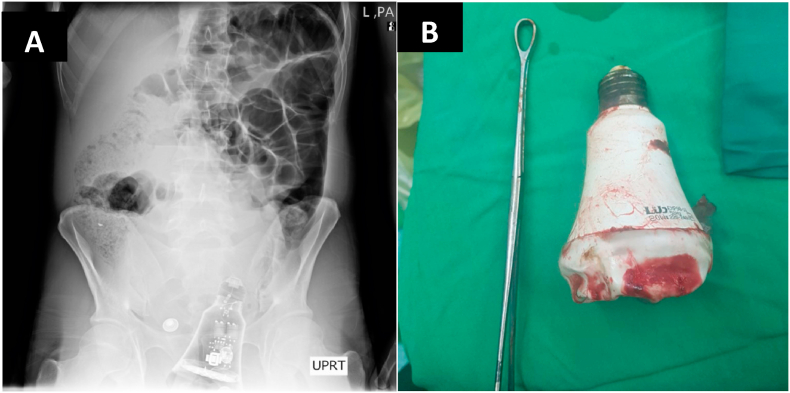
The Lamp as a foreign body in the rectum (A), and after removal (B).

A 48-year-old man was admitted to the general surgery department after referring from the forensic ward of the hospital. Two days prior to hospitalization, the patient was raped following a conflict. There was no remarkable history of sexual dysfunction and/or psychosocial history. The patient was complaining of severe pain in the lower abdomen and painful and very difficult deification. On examination, deep abrasions around the anus and severe tenderness in the hypogastric region were observed. Following the pain radiographic (Fig. 3), the patient took to the operating room. To remove the glass bottle via speculum was unsuccessful, laparotomy was performed under general anesthesia (Midazolam, Sufentanil, Poopofol, and Cisatracurium were administered for conventional induction. Meanwhile, Noradrenaline was injected an initial infusion of 0.03 μg/Kg/Min during induction to inhibit the peripheral vascular dilation induced by general anesthetics. Propofol and Remifentanil were used for intraoperative maintenance). The object was removed by colotomy of the sigmoid area and the colon was initially restored.

*In summary,* all the patients were referred to Imam Hossein Hospital in Tehran, and the surgeries were performed by an attending general surgeon. In all three patients, there were no significant history of surgical, family, and pharmacological. All the patients were males. On average, patients were referred to the hospital 2 days after foreign body insertion (Table .1). The most common symptoms were hypogastric pain and the inability to defecate. On initial examination, the most common sign was brief tenderness in the hypogastric region. Evidence of abrasion around the anus was also found in all patients. None of the patients had a history of sexual dysfunction or psychiatric illness. The diagnosis was made in all 3 patients with a history and abdominal x-ray. All three patients were taken to the operating room in 2 patients the foreign body was removed under sedation with the help of a speculum and pliers. Recto-sigmoidoscopy was normal after extraction. In one patient, an attempt to remove a foreign body from the anus was unsuccessful and a laparotomy was performed. The Two first cases were discharged the day after surgery and the third case has been discharged following three days of the hospitalization. All the patients were referred to physiological counseling.

**Table 1:** The table shows the characteristics of all three patients.

Age	Sex	Physical exam	Type of FB	X-ray	Operation	Cause	Hospital stay/duration
43	Male	Mild tenderness in lower abdomen	water hose	Fig. 1	Trans anal removal	Sexual violence	1/day
40	Male	Mild tenderness in lower abdomen + abrasion around the anus	glass bottle	Fig. 2	Trans anal removal	Sexual violence	1/day
48	Male	Mild tenderness in lower abdomen and deep abrasion around the anus	lamp	Fig. 3	Laparotomy and colotomy	Sexual violence	5/days

## 3 Discussion

Human nature is inherently opposed to any kind of force and violence; therefore, any use of violence is regarded as deviation. Insertion of foreign body in the rectum is a commonly encountered situation in clinical practice these are mostly inserted through the rectum and occasionally, few orally ingested foreign bodies may get impacted in the rectum. A variety of objects have been reported in the literature. Two-thirds of the patients are males in their 30s or 40s, who use such objects for autoerotic purposes [3,9]. It is important to know the history of similar episodes and any psychosexual behavior abnormalities in such individuals a proper history and examination is mandatory to look for signs and symptoms of perforation and infection, such as fever, severe abdominal pain and bleeding [[10], [11], [12]]. Intentional dipping of foreign bodies through the rectum into the rectum and recto sigmoid for sexual gratification has become more common in the last two decades. Reports indicate plastic and glass soda bottles have been the most common foreign objects [13]. To determine the perforation by FBs is the first step in the management of FBs. If there is a perforation, it should be determined whether the patient is stable or not. In case clinical instability, the patient needs immediate resuscitation. In a stable patient, a Computed Tomography (CT) scan can determined if a rectal perforation occurred or not. When a FB is removed or absent in the rectum, rigid proctoscopy or endoscopic evaluation should be taken into account. Endoscopic evaluation may be a great help in revealing the rectal injures or the FB lodged higher in the rectum [4,9]. In stable patient with no signs of perforation, the RFBs can be removed *trans*-anal, if not, then a *trans*-abdominal approach is needed (as the third case), [14]. Rigid proctoscopy or flexible sigmoidoscopy is recommend after the rectal FB removal. Flexible endoscopy is reserved for objects that are located proximally in the rectum or the distal sigmoid colon. Endoscopy reveals an excellent view of the rectum mucosa, once the Fb removed successfully, the bowl mucosa should be evaluated by endoscopic approach [4]Survivors of sexual violence experience numerous short-term and long-term negative physical and mental health outcomes, including physical injury, sexually transmitted infections (STIs), unwanted pregnancy, unsafe abortion, anxiety, shame, posttraumatic stress, and depression [15]. Death from sepsis and multi system organ failure has been reported. Damage to the anal sphincter may result in mild to severe fecal incontinence, bleeding from lacerations of rectal mouse are usually self-limited [4].

## 4 Conclusion

Due to the private nature of sexual violence, it is difficult to estimate the extent of the problem. Sexual violence has a profound effect on the health of body and mind. At the same time it causes physical injuries, the treatment of these patients should include physical and mental therapies at the same time. The evaluation of the patients with rectal foreign bodies need to be done in an orderly manner, with appropriate examination, laboratory and radiographic evaluation. In stable, non-perforated patients, tans-anal approach under sedation is a good approach. If it fails, the patient needs to go to operating room for further anesthetic and surgical interventions.

## Ethical approval

This is a case report paper.

## Sources of funding

None.

## Author contribution

AMT, SS and BN conceived and designed the study; PQ and FN wrote the manuscript; JZB and SS helped collect data; PQ confirmed the eligibility of the participants' for the study; FN and JZB Supervised the whole study and approved the final version of the manuscript.

## Registration of research studies

Not applicable.

## Guarantor

Javad zebarjadi Bagherpour, the corresponding author, accepted full responsibility for the work and/or the conduct of the study, had access to the data, and controlled the decision to publish.

## Provenance and peer review

Not commissioned, externally peer-reviewed.

## Consent

Informed consent was obtained from all the three patients, for publication of this case report and accompanying images. A copy of the written consent is available for review by the Editor-in-Chief of this journal on request.

## Declaration of competing interest

The authors report no declarations of interest.
